# Zinc oxide and indium-gallium-zinc-oxide bi-layer synaptic device with highly linear long-term potentiation and depression characteristics

**DOI:** 10.1038/s41598-022-05150-w

**Published:** 2022-01-24

**Authors:** Hyun-Woong Choi, Ki-Woo Song, Seong-Hyun Kim, Kim Thanh Nguyen, Sunil Babu Eadi, Hyuk-Min Kwon, Hi-Deok Lee

**Affiliations:** 1 Department of Electronics Engineering, Chungnam National University, 99, Daehak-ro, Yuseong-gu, Daejeon, Republic of Korea; 2 Department of Semiconductor Processing Equipment, Semiconductor Convergence Campus of Korea Polytechnic College, 41-12, Songwon-Gil, Kongdo-Eup, Anseong, Kyunggi-Do Republic of Korea

**Keywords:** Electrical and electronic engineering, Long-term memory

## Abstract

The electrical properties, resistive switching behavior, and long-term potentiation/depression (LTP/LTD) in a single indium-gallium-zinc-oxide (IGZO) and bi-layer IGZO/ZnO (ZnO: zinc oxide) memristors were investigated for synapse application. The use of the oxide bi-layer memristors, in particular, improved electrical properties such as stability, memristor reliability, and an increase in synaptic weight states. The set voltage of bi-layer IGZO/ZnO memristors was 0.9 V, and the reset voltage was around − 0.7 V, resulting in a low-operating voltage for neuromorphic systems. The oxygen vacancies in the X-ray photoelectron spectroscopy analysis played a role in the modulation of the high-resistance state (HRS) (oxygen-deficient) and the low-resistance state (oxygen-rich) region. The V_RESET_ of the bi-layer IGZO/ZnO memristors was lower than that of a single IGZO, which implied that oxygen-vacancy filaments could be easily ruptured due to the higher oxygen vacancy peak HRS layer. The nonlinearity of the LTP and LTD characteristics in a bi-layer IGZO/ZnO memristor was 6.77% and 11.49%, respectively, compared to those of 20.03% and 51.1% in a single IGZO memristor, respectively. Therefore, the extra ZnO layer in the bi-layer memristor with IGZO was potentially significant and essential to achieve a small set voltage and a reset voltage, and the switching behavior to form the conductive path.

## Introduction

In complex situations, the human brain can process information in parallel and accurately recognize objects and visual information^1,2^. Neuromorphic information processing systems are expected to be the next-generation computing technology that can overcome the limitations of traditional information processing systems, such as von Neumann computing. The use of memory structures is the most notable distinction between neuromorphic systems and traditional information processing systems. Several transistors and capacitors have been used in conventional information processing systems to emulate a single synapse, increasing power consumption, and limiting integration density^3–5^. Many kinds of research are currently being studied to develop a single device with a new concept that simultaneously performs inherent learning and data storage functions without memory devices^6–11^. Because of its ability to modulate conductance, resistive switching random access memory (ReRAM) has recently emerged as the most promising candidate for synaptic devices. Furthermore, because of its simple structure, fast switching speed, lower power consumption, and higher scalability, ReRAM with a metal–insulator–metal structure capable of realizing synaptic networks have an advantage of integration at high density (4F^2^)^12–14^.

Among the various dielectric materials used as resistive switching (RS) layers, such as hafnium oxide^15,16^, tantalum oxide^17,18^ and titanium oxide^19–21^, indium-gallium-zinc-oxide (IGZO) and zinc oxide (ZnO) have attracted much attention as one of the most promising due to its outstanding good uniformity for large area deposition, low cost, and multiple functional abilities^22,23^. Furthermore, an advantage of IGZO material is the ease with which the RS behavior can be switched as the mobility of the carrier increases due to an increase in indium concentration, allowing for high-speed operation. Following a high driving current, the increased concentration of indium in the IGZO layer increased mobility, while the gallium concentration controlled the amount of oxygen vacancy^24,25^. ZnO is an excellent material of chemical stability, and has a wide direct bandgap and high transparency properties. However, because donor defects in ZnO materials contribute to n-type conduction, high resistive ZnO switching films are difficult to achieve. The low-resistance state (LRS) layer is positioned on top of the bi-layer structure, resulting in good switching behavior. However, memristor devices for hardware-based neural networks should consider the linear and symmetric changes in conductance with the number of potentiation and depression pulses. Furthermore, the SET pulse applied as the pre-synaptic input can determine the consumed energy per the weight update for the network training. The relatively high SET amplitudes and long pulse width must be further improved for application in energy-efficient and largescale neuromorphic device arrays. The potentiation and depression characteristics of single-layer memristor devices are frequently nonlinear, resulting in less efficient neural network processing. It is believed that the bi-layer memristor device’s structure achieves high linearity of the potentiation and depression characteristics. The electrical properties of the ReRAM with an additional oxide layer between the bottom electrode and the IGZO layers, on the other hand, are yet to be investigated. The use of oxide bi-layers improved electrical properties such as stability, memristor reliability, and an increase in synaptic weight states^26–28^.

This paper proposed bi-layer IGZO/ZnO memristors to improve the electrical characteristics and synaptic linearity in long-term potentiation/depression (LTP/LTD) characteristics compared with a single IGZO memristor. The conduction mechanism of the charge transport behavior at HRS and LRS was validated by the role of the potential barrier between the bottom electrode and HRS material. To our knowledge, the high linearity in LTP/LTD characteristics of the bi-layer IGZO/ZnO memristors is more linear than that of other reported devices^29–32^.

## Methods

**Figure 1:**
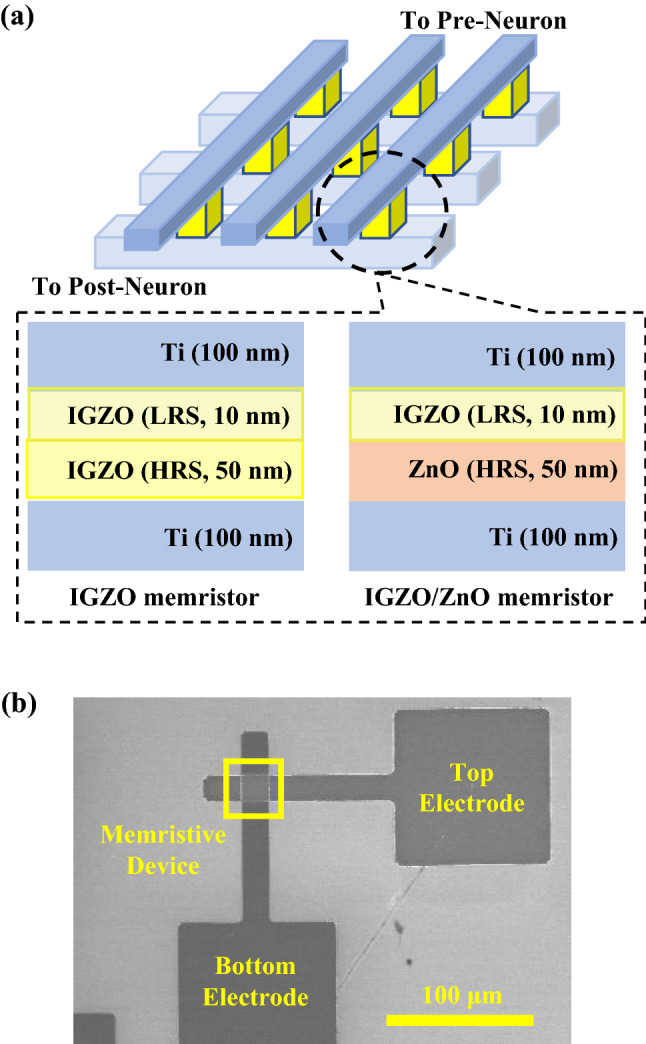
(**a**) A schematic of crossbar structure in a single IGZO and bi-layer IGZO/ZnO memristors (IGZO: Indium-gallium-zinc-oxide, ZnO: Zinc Oxide), (**b**) top-view scanning electron microscopy images of the crossbar structure for the memristor device.

A single structure of memristor crossbar was fabricated to a square type, as shown in Fig. 1, which could be integrated at high density. The Ti layer, which served as the bottom electrode, was deposited at 100 nm using a radio frequency (RF) sputtering system and patterned using photolithography’s lift-off process. Memristors are classified into two types: single IGZO memristors and bi-layer IGZO/ZnO memristors. The single-layer IGZO was deposited using the following RF sputtering system. First, 50 nm-thick IGZO used as an HRS was deposited with the molecular composition of In:Ga:Zn = 1:1:1 at a working pressure of 2 mTorr (Ar gas flow rate: 30 standard cubic centimeters per min (sccm)) without substrate heating (substrate temperature < 50 °C). The 10 nm-thick IGZO for an LRS was then deposited with the molecular composition In:Ga:Zn = 2:2:7, and the other conditions were the same as described above. To control the amount of oxygen vacancy, the oxygen gas flow was adjusted as 20 sccm for HRS and 1 sccm for LRS when the IGZO layers were deposited. For the bi-layer IGZO/ZnO structure, a 50 nm ZnO layer was deposited using an atomic layer deposition system with DEZn (diethylzinc, Zn(CH_2_CH_3_)_2_) and an H_2_O of source precursors at an 80 °C chamber temperature, followed by a 10 nm IGZO LRS deposition using an RF sputtering system. Finally, the Ti layer was deposited as the top electrode using the RF sputtering system, which is identical to the bottom electrode process through photolithographic patterning to form a crossbar type device. Figure 1b showed the top-view images of the field emission scanning electron microscopy (FE-SEM, Hitachi, Japan, S-4800) in a 20 μm × 20 μm crossbar structure.

The electrical properties of a single IGZO and bi-layer IGZO/ZnO memristors were measured at room temperature using the Keysight B1500A semiconductor device analyzer. For the transition from HRS to LRS, the current compliance was used. The Keysight B1530 waveform generator/fast measurement unit, which also has current and voltage measurement functions, was used to apply voltage pulses across the single IGZO and bi-layer IGZO/ZnO memristors. Precision and long-term sampling measurements were used to measure the current across the diffusive memristor and to measure the voltage at the output of the waveform generator. The long-term sampling measurements allowed the application of pulse signals down to 100 ns and precise current measurement at a sampling rate of 50 ns. To minimize the disturbance on device conductance, a write pulse with amplitude 2 V was applied across a single IGZO and bi-layer IGZO/ZnO memristor, and then a short duration (20 or 50 ms) was used as the read voltage. Positive/negative pulses were applied at a rate of 50 for single IGZOs and 100 for bi-layer IGZO/ZnO memristors, and the current was measured after each stimulation pulse. All equipment in the setup is controlled by the Agilent Easy Expert software.

## Results and discussion

**Figure 2:**
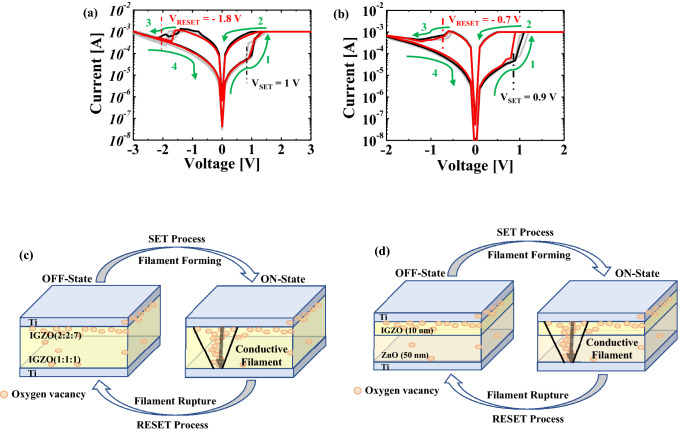
I-V typical bipolar switching behavior of (**a**) a single IGZO and (**b**) bi-layer IGZO/ZnO memristors, and the switching behavior of (**c**) a single IGZO and (**d**) bi-layer IGZO/ZnO memristors.

Figure 2 shows the RS behaviors of 10 cycles in a single IGZO and bi-layer IGZO/ZnO memristors. This result shows the typical bipolar RS characteristics with the bottom electrode grounded in electrical measurements. The memristor devices are initially in HRS mode. A voltage sweep from 0 to 3 V (meaning arrow “1” in the figure) is applied to the top electrode with a compliance current of 1 mA, and the RS behaviors of the single IGZO and bi-layer IGZO/ZnO memristors change from HRS to LRS. The compliance current is set to prevent memristor devices from being permanently damaged by a sudden increase in current levels. Once again, the voltage sweep is applied from 2 to 0 V (meaning arrow “2” in the figure) to measure the current and the RS behaviors of the single IGZO and bi-layer IGZO/ZnO memristors that remain in the LRS and show a high current level. The arrows “1” and “2” in the figure are referred to as “SET,” and they indicate that the RS behaviors of the single IGZO and bi-layer IGZO/ZnO memristors change from HRS to LRS. The process of changing from arrow “3” to “4” in the figure is called “RESET,” and it means that the RS behaviors of the single IGZO and bi-layer IGZO/ZnO memristors change from LRS (arrow “3”) to HRS (arrow “4”). Because conductance values (the inverse of resistance values) are used as synaptic weights in binarized neural networks, these findings can help them. The set voltages of the single IGZO and bi-layer IGZO/ZnO memristors are 1 V and 0.9 V, respectively. In contrast, the reset voltages of a single IGZO and bi-layer IGZO/ZnO memristors are about − 1.8 V and − 0.7 V, respectively. Therefore, in the case of IGZO/ZnO structure, the memristor device can be used as a low operation, which can improve the RS speed because the donor defects in ZnO materials contribute to the n-type conduction.

**Figure 3:**
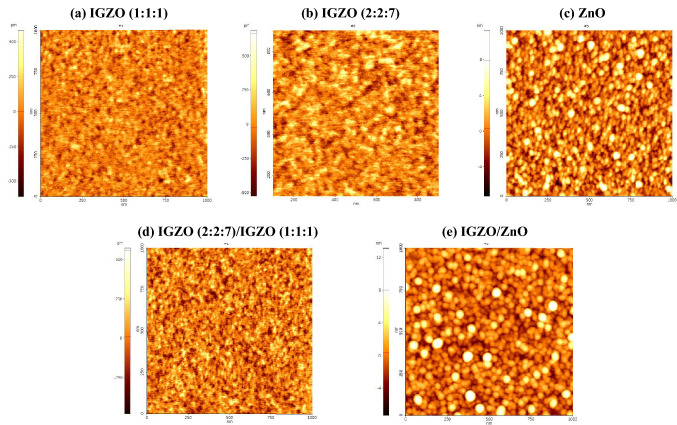
Atomic force microscopy topography images of single (**a**) IGZO (1:1:1), (**b**) IGZO (2:2:7), and (**c**) ZnO films, and (**d**) a single IGZO memristor(IGZO(2:2:7)/IGZO(1:1:1)) and (**e**) a bi-layer IGZO/ZnO memristor. ZnO film can be observed by small grains of nanometric dimension.

**Figure 4:**
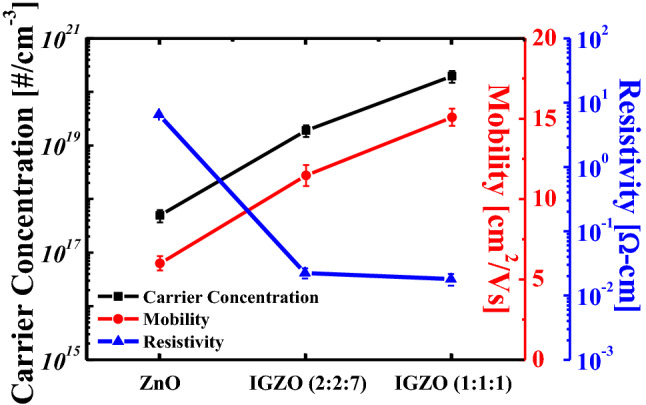
Hall measurements on the carrier concentration, resistivity, and Hall mobility of a single IGZO (1:1:1), IGZO (2:2:7), and ZnO films.

**Figure 5:**
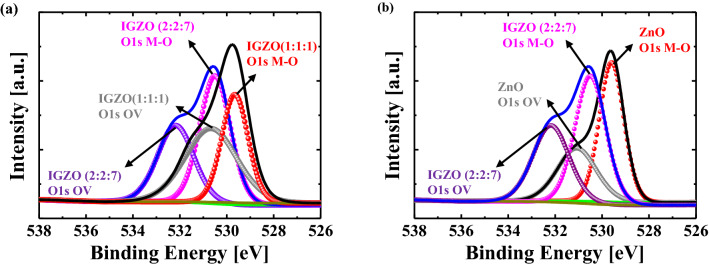
X-ray photoelectron spectroscopy spectra of O1s (**a**) a single IGZO and (**b**) bi-layer IGZO/ZnO memristors. The proportions of the oxygen vacancy peaks (O1) of the high-resistance state for a single IGZO and bi-layer IGZO/ZnO memristors are 45.2% and 38.2%, respectively, while that of LRS for both memristors is about 43.4%.

Figure 3 shows the atomic force microscopy results of a single IGZO (1:1:1), IGZO (2:2:7), and ZnO films, and bi-layer memristors for a measurement area of 1 × 1 μm. The root-mean-square (RMS) roughness value of a single IGZO (1:1:1), IGZO (2:2:7), and ZnO films is 0.112 nm, 0.145 nm, and 2.247 nm, respectively. The RMS roughness of a single IGZO memristor and a bi-layer IGZO/ZnO memristor is 0.121 nm and 2.176 nm, respectively. These findings imply that small grains with a nanometric dimension of 33 can be used to observe a typical polycrystalline ZnO film. Figure 4 depicts the results of the Hall measurements on carrier concentration, resistivity, and Hall mobility of a single IGZO (1:1:1), IGZO (2:2:7), and ZnO film. The electrical properties of the carrier concentration, resistivity, and Hall mobility for a single IGZO (1:1:1) and IGZO (2:2:7) films are greater than for a single ZnO film^33,34^. In particular, the resistivity of IGZO (2:2:7) decreases with the increase of gallium (Ga) content because Ga^3+^ forms strong bonds with oxygen^33,34^. The resistivity of each layer for a single IGZO and bi-layer IGZO/ZnO memristors is proportional to the reciprocal of the product of carrier concentration *N* and mobility *μ* as reported in Ref.^35^. It can confirm the conjecture of controlling the carrier concentration via oxygen-related defects associated with Ga. X-ray photoelectron spectroscopy (XPS, Thermo Fisher Scientific, USA, K-Alpha^+^) measurements were performed to investigate the chemical composition of the single IGZO and bi-layer IGZO/ZnO memristors, to verify the proportions of the oxygen vacancy. Figure 5 depicts the XPS analysis result of the oxygen vacancy peak (O1) spectra in the surface after deposition of each single HRS and LRS layer for the single IGZO and bi-layer IGZO/ZnO memristors using the Gaussian peak fitting. The proportions of the O1 of the HRS layer for a single IGZO and bi-layer IGZO/ZnO memristors are 45.2 percent and 38.2 percent, respectively, while the proportion of the LRS layer for both memristors is around 43.4 percent. Because the Ga/Zn ratio determines the oxygen concentration in HRS and LRS layers during IGZO sputter-deposition, increasing the Ga/Zn ratio increases the number of non-oxygen vacancies in the memristor devices, resulting in a lower conductivity layer^36^. The chemical composition obtained from the XPS analysis result plays an important role in distinguishing each HRS and LRS layer for the RS performance in the bi-layer memristor structure. The oxygen vacancies modulate the HRS layer (oxygen-rich) into the LRS layer (oxygen-deficient). The V_RESET_ of the bi-layer IGZO/ZnO memristors is lower than that of a single IGZO, which implied that the oxygen vacancy filaments could be easily ruptured due to the lower oxygen vacancy peak HRS layer.

**Figure 6:**
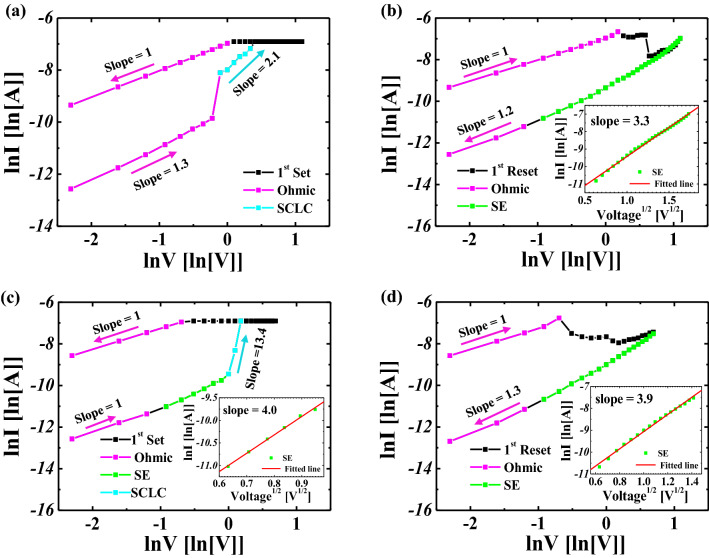
Analysis of the conduction mechanism corresponding to SET and RESET (**a**), (**b**) a single IGZO memristor and (**c**), (**d**) bi-layer IGZO/ZnO memristor, respectively. Schottky emission mechanism is observed in the single IGZO and bi-layer IGZO/ZnO memristors at the RESET, which can be attributed to the interface barrier.

To verify the mechanism of the RS behaviors for a single IGZO and bi-layer IGZO/ZnO memristors, the corresponding I–V characteristics of the SET and RESETs are plotted in Fig. 6. A linear fitting slope based on experimental data for the single IGZO and bi-layer IGZO/ZnO memristors is close to one, indicating a linear relationship between the current and applied voltage^37^. The charges originating at the metal electrode interface are thought to be trapped by the HRS layer’s empty trap sites of IGZO and ZnO. As the electric field across memristor devices increases, the steep current for a single IGZO is followed by a quadratic term (I ∝ V^2^) with the increase of the injected charges when the conductive filaments form between two electrodes, as shown Fig. 6. When the empty trap sites are gradually occupied completely, the slope of the fitting line decreases by about 2, indicating that the conduction enters the trap-free space charge limited current (SCLC). It implies that the SCLC is dominant because the majority of injected electrons contribute to the current component^38–44^.

However, the slope of the fitting line at the high electric field for bi-layer IGZO/ZnO memristor is found to be 4.0, which means that the Schottky emission mechanism is dominant. In a high electric field, the Schottky emission mechanism may be caused by oxygen vacancies near the metal/metal-oxide interface. The Schottky emission and ohmic mechanisms dominate the I–V characteristics of the RESET, as illustrated in Fig. 6. The switching behaviors of a single IGZO and bi-layer IGZO/ZnO memristors were controlled by the interface properties due to the Schottky emission mechanism. It should be noted that the SCLC is the primary conduction mechanism in the SET for single-layer IGZO and bi-layer IGZO/ZnO memristors. We conclude the Schottky emission mechanism observed in a single IGZO and bi-layer IGZO/ZnO memristors at the RESET is attributed to the interface barrier.

**Figure 7:**
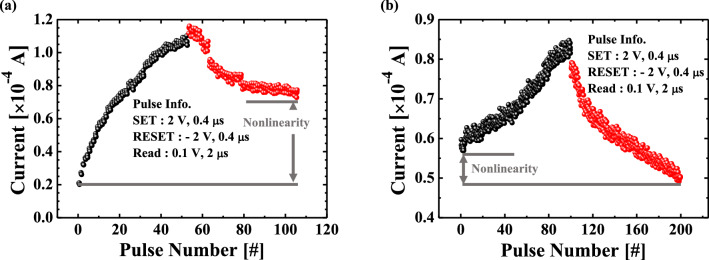
Long-term potentiation (LTP) and depression (LTD) characteristics of (**a**) single IGZO and (**b**) bi-layer IGZO/ZnO memristors. The applied positive/negative pulses were 50 for a single IGZO and 100 for bi-layer IGZO/ZnO memristors, respectively, and the current was read after each stimulation pulse was applied. The linearity and symmetricity of the LTP and LTD characteristics for bi-layer IGZO/ZnO memristor show more improved linearity than that for a single IGZO memristor.

Figure 7 shows the long-term potentiation/depression (LTP/LTD) characteristics with applied positive/negative pulses for an amplitude of 2 V in the two memristors. The positive (2 V, 400 ns) or negative (− 2 V, 400 ns) voltage pulses with the interval time (4.5 μs) are applied on the memristor devices, and then, the current is measured by a reading voltage pulse (0.2 V, 1 μs) after each pulse. Depending on the input spiking signal, the LTP and LTD characteristics exhibit gradual potentiation and depression in synaptic weight, which can be used to determine whether memristors can learn or not. When a potentiating input signal train of positive pulses with an amplitude of 2 V is applied to the top Ti metal of the memristor synapse (pre-neuron), the synaptic weight changes progressively as the current increases, indicating that oxygen vacancies are injected into the RS layer, and this process is then formed between TE and BE for potentiation. It can emulate the potential of oxygen vacancies for neuromorphic computing, which enhances the synapse weight by releasing neurotransmitters. When a depressing input signal train consisting of negative pulses with an amplitude of − 2 V is applied to the top metal, the synaptic weight is gradually depressed, and the conductive path formed by the oxygen vacancies moves away from the bottom metal, causing the current to decrease. The nonlinearity for the memristor devices is quantitatively given by Eq. (1)

1 $$Nonlinearity = average\left[ {\left| {\frac{{G - G_{Linear} }}{{G_{Linear} }}} \right| \times 100 \% } \right]$$

where G is the change in the conductance of memristive devices (equivalently, synaptic weight), and G_Linear_ is the linear change in conductance (determining training accuracy^45^).

**Figure 8:**
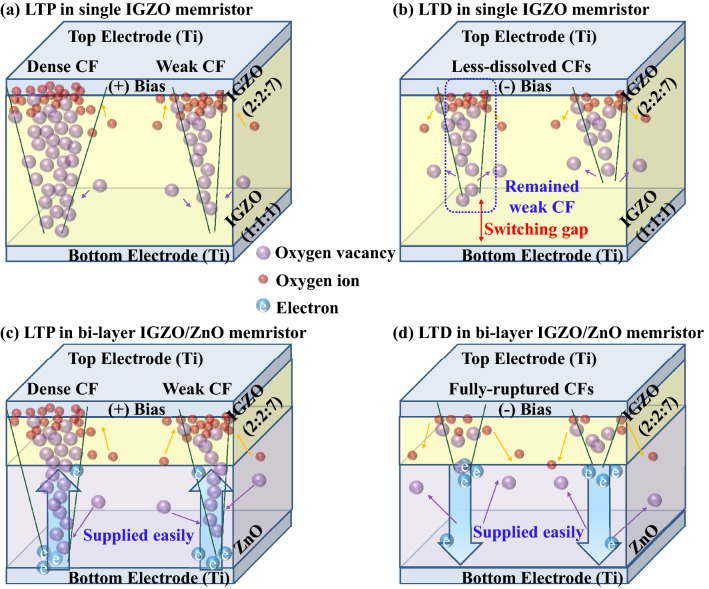
Schematic illustration of the transition mechanism from conductive filamentary switching for the single IGZO and bi-layer IGZO/ZnO memristors when evaluating synaptic devices corresponding to the LTP case and LTD case. (**a**), (**b**) a single IGZO, and (**c**), (**d**) bi-layer IGZO/ZnO memristors.

The nonlinearity of LTP and LTD characteristics in the bi-layer IGZO/ZnO memristor is 6.77% and 11.49%, respectively, while these are for in a single IGZO memristor is 20.03% and 51.1%, respectively^46^. When the number of synaptic weight states and total plasticity can be increased, oxygen vacancies closed to the metal/metal-oxide interface at the bottom electrode in the bi-layer IGZO/ZnO memristor are largely generated, appreciably increasing the effective Schottky barrier height as discussed in Fig. 7. As shown in Fig. 7, donor defects in ZnO materials that contributed to the n-type conduction are expected to have a more linear conductance response during LTD (b). The transition mechanism from conductive filamentary switching for a single IGZO and bi-layer IGZO/ZnO memristors is shown in Fig. 8, implying that the mobility difference between the two materials in the bi-layer structure, as discussed in Fig. 4, is critical. Therefore, the high electron conductivity of the ZnO layer in the bi-layer IGZO/ZnO memristor plays an important role in charge carriers to be injected easily under a small set voltage and a reset voltage switching behavior to form the conductive path. Not only the nonlinearity but also the asymmetry ratio is a critical factor in determining the learning accuracy^47^. The symmetric weight update between LTP and LTD in the memristor devices is defined by,

2 $$Asymmetry \,\,ratio = \frac{{G_{Potentiation} @ \left( {N_{x} } \right)}}{{G_{Depression} @ \left( {N_{max} - N_{x} } \right)}}$$

where G is the same as Eq. (1), N_max_ is total pulse number between G_min_ and G_max_, and N_x_ is a certain pulse number, indicating asymmetry ratio is “1” in ideal case^48^.

**Figure 9:**
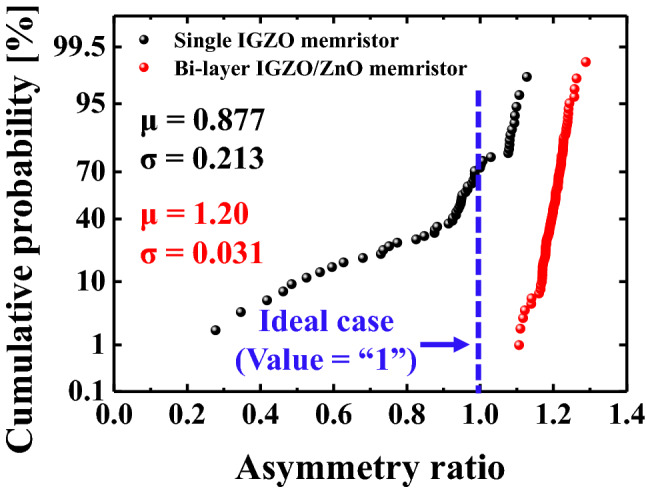
Asymmetry ratio distribution of a single IGZO and bi-layer IGZO/ZnO memristors. The standard deviation of bi-layer IGZO/ZnO memristors shows much more improvement compared to that of a single IGZO memristor, meaning close to ideal synapse device.

Figure 9 shows the cumulative probability results of asymmetry ratios for the single IGZO and bi-layer IGZO/ZnO memristors. The average asymmetry ratio for a single IGZO memristor and a bi-layer IGZO/ZnO memristor is 0.877 and 1.20, respectively. The standard deviations normalized to the mean ratio for two memristors, on the other hand, are 0.213 and 0.031, respectively. As a result, the comprehensive nonlinearity and asymmetry results are shown in Table 1, indicating that a bi-layer IGZO/ZnO memristor made by inserting a ZnO layer for the HRS layer is more suitable for ideal synaptic devices. It has the potential to be a desirable option to be used in implementing neural networks in the future.

**Table 1:** Comprehensive summary table of DC and LTP/LTD characteristics in the single IGZO and bi-layer IGZO/ZnO memristors.

Parameters	Single IGZO memristor	Bi-layer IGZO/ZnO memristor	Ideal case	Ref
Voltage (Vset/Vreset)	1–1.8 V	0.9–0.7 V	–	0.71 V/−0.74 V^49^
NL (in LTP)	21.03%	6.77%	0%	^45^
NL (in LTD)	51.1%	11.49%	0%	^45^
Asymmetry ratio	Refer to Fig. 9		1	^48^

## Conclusion

We investigated the electrical characteristics, RS behavior, and the LTP/LTD of ZnO and IGZO and bi-layer memristors for high-performance synaptic devices. The set and reset voltages of the bi-layer IGZO/ZnO memristors are 1 V, and − 0.7 V, achieving low operating voltage to realize the neuromorphic systems. The oxygen vacancies played a role in the modulation of the HRS layer (oxygen-deficient) and the LRS layer (oxygen-rich) region. The V_RESET_ of the bi-layer IGZO/ZnO memristors was lower than that of a single IGZO, which implied that the oxygen vacancy filaments could be easily ruptured due to the higher oxygen vacancy peak in the HRS layer. When compared to a single IGZO memristor, the nonlinearity and asymmetry ratios of the LTP and LTD characteristics for the bi-layer IGZO/ZnO memristor improved significantly. These findings were significant because they revealed the difference in mobility between two materials in a bi-layered structure. As a result, the ZnO layer’s role in the bi-layer IGZO/ZnO memristor was potentially significant and necessary for charge carriers to be injected easily under a small set voltage and a reset voltage, as well as the switching behavior to form the conductive path.

## Data Availability

The datasets generated during and/or analyzed during the current study are available from the corresponding author on reasonable request.

## References

[1] Hebb DO (2005). The organization of behavior: a neuropsychological theory.

[2] Gerstner W, Ritz R, Van Hemmen JL (1993). Why spikes? Hebbian learning and retrieval of time-resolved excitation patterns. Biol. Cybern..

[3] Indiveri G, Chicca E, Douglas R (2006). A VLSI array of low-power spiking neurons and bistable synapses with spike-timing dependent plasticity. IEEE Trans. Neural Netw..

[4] Hahnloser RH, Sarpeshkar R, Mahowald MA, Douglas RJ, Seung HS (2000). Digital selection and analogue amplification coexist in a cortex-inspired silicon circuit. Nature.

[5] Diorio C, Hasler P, Minch A, Mead CA (1996). A single-transistor silicon synapse. IEEE Trans. Electron Devices.

[6] Yakopcic C, Taha TM, Subramanyam G, Pino RE, Rogers S (2011). A memristor device model. IEEE Electron Device Lett..

[7] Lanza M (2019). Recommended methods to study resistive switching devices. Adv. Electron. Mater..

[8] Beck A, Bednorz J, Gerber C, Rossel C, Widmer D (2000). Reproducible switching effect in thin oxide films for memory applications. Appl. Phys. Lett..

[9] Liu S, Wu N, Ignatiev A (2000). Electric-pulse-induced reversible resistance change effect in magnetoresistive films. Appl. Phys. Lett..

[10] Kim SG, Han JS, Kim H, Kim SY, Jang HW (2018). Recent advances in memristive materials for artificial synapses. Adv. Mater. Technol..

[11] Mohammad B (2016). State of the art of metal oxide memristor devices. Nanotechnol. Rev..

[12] Burr GW (2017). Neuromorphic computing using non-volatile memory. Adv. Phys. X.

[13] Waser R, Aono M (2007). Nanoionics-based resistive switching memories. Nat. Mater..

[14] Yoshida C, Tsunoda K, Noshiro H, Sugiyama Y (2007). High speed resistive switching in Pt∕ TiO_2_∕ Ti N film for nonvolatile memory application. Appl. Phys. Lett.

[15] Syu Y-E (2013). Atomic-level quantized reaction of HfO_x_ memristor. Appl. Phys. Lett..

[16] Wang H (2019). Bio-inspired synthesis of mesoporous HfO_2_ nanoframes as reactors for piezotronic polymerization and Suzuki coupling reactions. Nanoscale.

[17] Kim S (2015). Experimental demonstration of a second-order memristor and its ability to biorealistically implement synaptic plasticity. Nano Lett..

[18] Li X (2016). Electrode-induced digital-to-analog resistive switching in TaO_x_-based RRAM devices. Nanotechnology.

[19] Seo K (2011). Analog memory and spike-timing-dependent plasticity characteristics of a nanoscale titanium oxide bilayer resistive switching device. Nanotechnology.

[20] Gao L (2015). Fully parallel write/read in resistive synaptic array for accelerating on-chip learning. Nanotechnology.

[21] Park J (2016). TiOx-based RRAM synapse with 64-levels of conductance and symmetric conductance change by adopting a hybrid pulse scheme for neuromorphic computing. IEEE Electron Device Lett..

[22] Fan Y-S, Liu P-T, Hsu C-H (2013). Investigation on amorphous InGaZnO based resistive switching memory with low-power, high-speed, high reliability. Thin Solid Films.

[23] Chen M-C (2010). Influence of electrode material on the resistive memory switching property of indium gallium zinc oxide thin films. Appl. Phys. Lett..

[24] Kimizuka N, Yamazaki S (2016). Physics and technology of crystalline oxide semiconductor CAAC-IGZO: fundamentals.

[25] Kim M-S (2012). Effects of the oxygen vacancy concentration in InGaZnO-based resistance random access memory. Appl. Phys. Lett..

[26] Kim S (2018). Engineering synaptic characteristics of TaO_x_/HfO_2_ bi-layered resistive switching device. Nanotechnology.

[27] Wang J, Zhuge X, Zhuge F (2021). Hybrid oxide brain-inspired neuromorphic devices for hardware implementation of artificial intelligence. Sci. Technol. Adv. Mater..

[28] Li J (2017). Tuning analog resistive switching and plasticity in bilayer transition metal oxide based memristive synapses. RSC Adv..

[29] Park, S.,* et al*. Neuromorphic speech systems using advanced ReRAM-based synapse. *IEEE International Electron Devices Meeting* (IEEE, 2013)

[30] Romero LP (2019). Training fully connected networks with resistive memories: impact of device failures. Faraday Discuss..

[31] Thakur CS (2018). Large-scale neuromorphic spiking array processors: a quest to mimic the brain. Front. Neurosci..

[32] Yu, S., *et al.* Scaling-up resistive synaptic arrays for neuro-inspired architecture: challenges and prospect. *IEEE International Electron Devices Meeting (IEDM)* (IEEE, 2015)

[33] Hsu C-M, Tzou W-C, Yang C-F, Liou Y-J (2015). Investigation of the high mobility IGZO thin films by using co-sputtering method. Materials.

[34] Zeng Y (2007). Study on the Hall-effect and photoluminescence of N-doped p-type ZnO thin films. Mater. Lett..

[35] Zan HW, Tsai WW, Chen CH, Tsai CC (2011). Effective mobility enhancement by using nanometer dot doping in amorphous IGZO thin-film transistors. Adv. Mater..

[36] Chai Z, Liu Y, Lu X, He D (2014). Reducing adhesion force by means of atomic layer deposition of ZnO films with nanoscale surface roughness. ACS Appl. Mater. Interfaces..

[37] Lim EW, Ismail R (2015). Conduction mechanism of valence change resistive switching memory: a survey. Electronics.

[38] Mondal S, Chueh C-H, Pan T-M (2014). Current conduction and resistive switching characteristics of Sm_2_O_3_ and Lu_2_O_3_ thin films for low-power flexible memory applications. J. Appl. Phys..

[39] Yu L-E (2008). Structure effects on resistive switching al/TiOx/al devices for RRAM applications. IEEE Electron Dev. Lett..

[40] Liu Q (2008). Resistive switching memory effect of Zr O_2_ films with Zr+ implanted. Appl. Phys. Lett..

[41] Peng H, Wu T (2009). Nonvolatile resistive switching in spinel ZnMn_2_O_4_ and ilmenite ZnMnO_3_. Appl. Phys. Lett..

[42] Lee H (2009). Low-power and nanosecond switching in robust hafnium oxide resistive memory with a thin Ti cap. IEEE Electron Device Lett..

[43] Chen C, Yang Y, Zeng F, Pan F (2010). Bipolar resistive switching in Cu/AlN/Pt nonvolatile memory device. Appl. Phys. Lett..

[44] Ismail M (2014). Forming-free bipolar resistive switching in nonstoichiometric ceria films. Nanoscale Res. Lett..

[45] Wang IT, Chang CC, Chiu LW, Chou T, Hou TH (2016). 3D Ta/TaO_x_ /TiO_2_/Ti synaptic array and linearity tuning of weight update for hardware neural network applications. Nanotechnology.

[46] Bae JH, Lim S, Park BG, Lee JH (2017). High-density and near-linear synaptic device based on a reconfigurable gated schottky diode. IEEE Electron Device Lett..

[47] Ielmini D (2018). Brain-inspired computing with resistive switching memory (RRAM): Devices, synapses and neural networks. Microelectron. Eng.

[48] Yu S (2017). Neuro-inspired computing using resistive synaptic devices.

[49] Min S-Y, Cho W-J (2021). High-performance resistive switching in solution-derived IGZO: N memristors by microwave-assisted nitridation. Nanomaterials.

